# Rotten and gold apples: inside and outside the gray zone of a ROC curve

**DOI:** 10.1186/s13054-023-04796-w

**Published:** 2024-01-12

**Authors:** Antonio Messina, Michelle Chew, Maurizio Cecconi

**Affiliations:** 1 Department of Biomedical Sciences, Humanitas University, Via Levi Montalcini 4, Pieve Emanuecle, Milan, Italy; 2 Department of Anesthesia and Intensive Care Medicine, IRCCS Humanitas Research Hospital, Via Manzoni 56, 20089 Rozzano, Milan, Italy; 3 Department of Anesthesia and Intensive Care, Biomedical and Clinical Sciences, Linköping University, Linköping, Sweden

**Keywords:** Hemodynamic monitoring, Fluid responsiveness, Fluid therapy, Pulse pressure variation, Prediction

## Dear Editor,

We were expecting some comment about the results of our paper, since this topic has been debated for years. In the words of George Bernard Shaw “If you have an apple and I have an apple (even rotten, we add) and we exchange these apples then you and I will still each have one apple. But if you have an idea and I have an idea and we exchange these ideas, then each of us will have two ideas”. So, ideas are always welcome and we read with interest the reply from Michard and colleagues [1].

The authors describe a well-known fact. The ROC curve for PPV and SVV and their positive and negative predictive values are nearly perfect under very specific conditions, i.e., in patients undergoing non-laparoscopic surgery with a closed chest and a tidal volume of 7–9 ml/kg (and without arrhythmias). In real life, however, these represent a minority of clinical situations. We agree that the underlying studies in our metanalysis do not represent ideal conditions, however they likely reflect real-life clinical heterogeneity and they speak to the effectiveness of PPV and SPV, rather than efficacy under ideal conditions. We therefore approached this problem pragmatically and rigorously by subdividing the studies and stratifying the results according to the surgical setting [2].

We concur with Dr. Michard and co-authors that the discriminatory value of PPV and SVV is likely to be higher in the subgroup of patients meeting all the conditions conducive to its reliable use—however this data was simply not available. To support this notion, we presented pooled AUCs among patients with a closed chest and abdomen, ventilated with > 8 ml/kg tidal volume in Table 4, demonstrating an AUC close to 0.9, even in prone patients.

Regarding changes in PPV after a functional hemodynamic test, we agree with the authors that this is an interesting but different primary research question; our research group is already working on it. We also agree that PPV has practical advantages since it does not require cardiac output monitoring. Our results even suggest that PPV may be more reliable than SVV. Functional hemodynamic tests are useful and research in this field should be encouraged.

Our meta-analysis is the most comprehensive and updated reality check of the accuracy of PPV and SVV in predicting fluid responsiveness during surgery. The perioperative use of PPV and SVV is still debated despite hundreds of publications over the last decades because of a simple and unavoidable point: simplifying any physiological interaction of the human body using a number (any number) may be effective only under strict and defined conditions. Ignoring this will only result in the comparison of (rotten) apples and pears.

Threshold values of PPV were already questioned more than a decade ago by Cannesson et al., who reported an overall PPV AUC of 0.89 (95% CI 0.86–0.92) in a cohort of 413 patients during general anesthesia and mechanical ventilation in four centers (still one of the biggest trial in this field). Importantly, the average tidal volume was 7.9 ± 1.3 ml/kg body weight (so, basically 8 ml/kg), the volume expansion consisted of 500 ml colloid solution (hetastarch 6% or modified fluid gelatin) given over 10–20 min, and the hemodynamic measurements were performed within 2–5 min after volume expansion [3]. All these variables (the amount of fluid, the rate of administration, the type of fluid used and the time points evaluating the effect of the fluid challenge) may impact on the binary 1/0 fluid responsiveness response. Accordingly, studying the PPV adopting the same tidal volume, but different fluid challenge tests will inevitably lead to different results. For instance, in a previous systematic review, we showed that in the last decade fluid challenges have been infused within shorter times, as compared to the past [4].

In our metanalysis, in the studies enrolling patients with closed chest and abdomen, the pooled AUC for PPV was 0.79 (95%CI 0.73–0.84) for a threshold of 10.9%, which is increased to 0.88 (0.82–0.93) in the studies ventilating the patients with a tidal volume of > 8 ml/kg. So, also when “physiologic limitations to their use are respected”, PPV reliability may be affected by other not-PPV related mechanisms.

Finally, we would like to stress another point. At a time of exponential progress in monitoring and data management supporting systems, such as AI augmented clinical decision making, one should consider a ROC curve of 0.79 positively. This is much better than guessing or tossing a coin. However, lessons learnt from recent research in fluid administration have demonstrated that it is unwise to make a decision based on a single variable. As precisely as Michard and colleagues suggest, decisions to administer fluids should be personalized by considering clinical grounds, the assessment of the adequacy of perfusion and by testing fluid responsiveness. This should be done regardless of whether ideal conditions are fulfilled. In other words, we should search for tests that matter for patients, and not for patients that matter for tests.

## Data Availability

Not applicable.

## Abbreviations

PPV: Pulse pressure variation
SVV: Stroke volume variation
ROC: Curve, receiving operating characteristics curve
AUC: Area under the ROC curve
